# Improved spin–orbit torque induced magnetization switching efficiency by helium ion irradiation

**DOI:** 10.1038/s41598-022-06960-8

**Published:** 2022-03-02

**Authors:** Suhyeok An, Eunchong Baek, Jin-A Kim, Ki-Seung Lee, Chun-Yeol You

**Affiliations:** 1 Department of Emerging Materials Science, DGIST, Daegu, 42988 Korea; 2 Emerging Materials Science Research Center, DGIST, Daegu, 42988 Korea

**Keywords:** Materials science, Physics

## Abstract

Increasing the efficiency of spin–orbit torque (SOT) is of great interest in spintronics devices because of its application to the non-volatile magnetic random access memory and in-logic memory devices. Accordingly, there are several studies to alter the magnetic properties and reduce the SOT switching current with helium ion irradiation, but previous researches are focused on its phenomenological changes only. Here, the authors observe the reduction of switching current and analyze its origins. The analyzed major reasons are improved spin Hall angle represented as the changed resistivity of heavy metal layer and the reduction of surface anisotropy energy at interface between heavy metal and ferromagnet. It is confirmed that almost linear relation between changed SHA and Pt resistivity by helium ion irradiation, which is attributed because of the increase in the scattering sources induced by structural distortion during ion penetration. From the calculated power consumption ratio based on the derived parameter, the requiring power decreases according to the degree of ion irradiation. Our results show that helium ion penetration induced layer and interfacial disturbance affects SOT induced magnetization switching current reduction and may provide possibility about helium ion irradiation based superior SOT device engineering.

## Introduction

Spin–orbit torque (SOT) induced magnetization switching is a perspective phenomenon to magnetic material based devices because of its potential application to the non-volatile magnetic random access memory (MRAM) and in-logic memory devices. SOT devices have two main benefits compare to the spin transfer torque (STT) MRAM. First, SOT switching is much faster (~ 1 ns) than STT-MRAM (~ 10 ns). Second, SOT-MRAM have reading path separated from the writing path, it is expected that more stable devices are possible and more margins in the reading and writing currents. In SOT switching, there are two essential ingredients: structural inversion symmetry breaking and strong spin–orbit coupling (SOC). A heavy metal (HM) / ferromagnetic metal (FM) bilayer structure satisfies those requirements. When charge current passes through the HM, the spin current is created inside of HM and injected into the FM by spin Hall effect (SHE)^1^ and/or the Rashba effect can create non-zero effective field at the interfaces^2^. For developing more effective SOT induced magnetization switching, many relevant parameters like spin Hall angle (SHA, *θ*_*SH*_)^3^, magnetic anisotropy field^4^ and Dzyaloshinskii-Moriya (DM) interaction^5^ are under the investigation. Among them, *θ*_*SH*_, a ratio of spin current to the charge current densities, is a vital parameter as indicators of converting efficiency from electric charge to spin current densities by SHE in HM. The *θ*_*SH*_ is one of the key players on SOT induced switching since the magnitude of SOT is proportional to the spin current. Therefore, *θ*_*SH*_ value has been investigated heavily in various HMs, like Ta (~ 0.15)^6^, β-W (~ 0.33)^7^ and Pt (~ 0.1)^8,9^.

Since the discovery of SOT, intensive efforts to enhance and control the strength of SOT are conducted by annealing^10^, resistivity control of HM layer^11^, normal metal (NM) insertion^12,13^, alloying^14^, interface modifying^15,16^, and ion irradiation^17,18^. Among them, the method of irradiating helium ion is known as leading a structural rearrangement while maintaining the overall atomic layer districts in the multi-stacked structure^19^ even if no ions remain in the sample due to the long penetration depth (> 50 nm)^20^. Ion irradiation induced structural reorganization affects layers and interfaces, resulting in the coercivity and anisotropy field change^21^, domain wall (DW) pinning site creation^22^, modulating DM Interaction^23^, and creating/guiding magnetic skyrmions^24^. This characteristic is also evident in the case of SOT, so it has been observed in various SOT relevant effects such as reduction of switching current by SOT^18^, influence on DW dynamics^25^, and multi-level state^26^. However, none of the previous studies about helium ion irradiation induced SOT modification have been paid attention in analyzing the HM quality caused by helium ion penetration. As *θ*_*SH*_ has a great influence on SOT induced phenomena and directly related with HM layer, it is important to verify both the interface and layer characteristics of the magnetic properties that contribute to the helium ion irradiation induced reduction of switching current.

Here, we confirm that helium ion irradiation reduced the SOT induced magnetization switching current, and conduct a dose dependent analysis of *θ*_*SH*_ through harmonic hall analysis, which was not previously analyzed, as well as interfacial effects such as PMA. We prepared Pt(5)/Co(0.8)/MgO(2) structure samples and irradiated helium ions in various doses. After helium ions irradiations, the critical current for SOT induced switching is measured under various in-plane field. We found that the switching current decreases 30.3% between dose 0 and 30 ions/nm^2^ at the in-plane field of 3.1 kOe. For understanding the decrease of switching current, we observed dose dependent tendency of the uniaxial anisotropy fields, and *θ*_*SH*_. The results show the decreasing tendency in anisotropy field (38.0% comparing with 0 and 30 ions/nm^2^) and increasing tendency in *θ*_*SH*_ (27.2% comparing with 0 and 30 ions/nm^2^). Furthermore, we measured the resistivities of single Pt layers according to dose and confirmed the relation between resistivity and *θ*_*SH*_. Based on our analysis, the change of *θ*_*SH*_ is caused by increased scattering sources by helium ion irradiation, resulting in almost linear improving of *θ*_*SH*_ according to variation of resistivity of Pt layer. From the calculated power consumption based on measured parameters, the requiring power reaches 87.4%, 59.6%, and 56.0% at dose of 10, 20 and 30 ions/nm^2^, respectively. We find that the SOT change due to helium ion penetration can be caused by the HM quality change as well as the interface. And it is expected that it may be possible to fabricate the more efficient SOT based device by modulating the layer and interface quality by helium ion irradiation.

## Results and discussion

### Sample preparation and coordinate information

**Figure 1:**
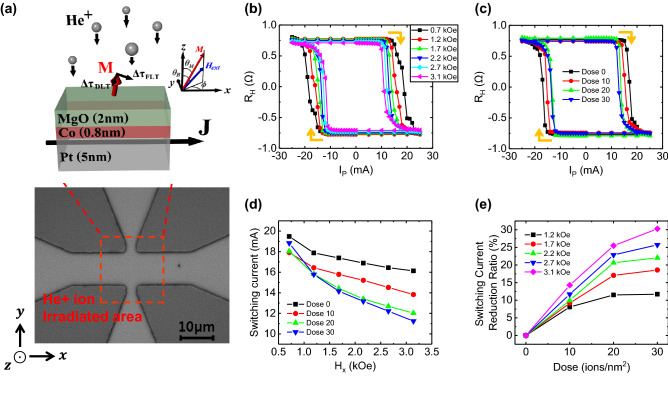
Schematic diagram and SOT induced magnetization switching results for various doses with the in-plane field. (**a**) Sample layer structure with the coordinate system and definitions of *θ*_*B*_, *θ*_*M*_ and *ϕ*. In bottom, optical image of Hall bar is shown and the helium ion irradiation area is depicted with red dotted rectangle. (**b**) SOT induced magnetization reversal is clearly shown by AHE Hall resistance loops for dose amount of 30 ions/nm^2^ sample. Additional in-plane magnetic field (0.7–3.1 kOe) is applied along the current line to break lateral symmetry. (**c**) SOT induced magnetization reversal loops for various doses with fixed external in-plane field of 2.2 kOe. (**d**) Critical switching current obtained from magnetization reversal loops as function of the external field for various doses. (e) Critical switching current reduction ratios compared with the pristine sample ($\left|\left({I}_{P.crit}-{I}_{P,crit}^{Dose 0}\right)/{I}_{P,crit}^{Dose 0}\right|\times 100 \%$) at each external field. Here, the error bars in (**d**) and (**e**) are smaller than symbol size.

We deposited Pt(5 nm)/Co(0.8 nm)/MgO(2 nm) heterostructure sample using magnetron sputter and patterned as 10 μm width Hall bar structure by photolithography technique. In Fig. 1a, the sample structure, coordinate systems, and helium ion irradiation area are depicted. *θ*_*M*_ is the polar angle between magnetization direction and z-axis, *θ*_*B*_ is the polar angle between the external magnetic field and z-axis, and *ϕ* is azimuthal angle from x-axis, and here we ignore the angle differences between the external magnetic field and magnetization directions in the azimuthal angle because of the negligible in-plane anisotropy. After Hall bar fabrication process, helium ion is irradiated in vertical direction with sample plane having acceleration energy of 30 keV, beam current of 5.5 pA, and dose from 0 to 30 ions/nm^2^ with step of 10 ions/nm^2^. Irradiation area covers whole Hall cross for avoiding signal mixing errors by signal differences between irradiation and non-irradiation area as shown red dotted rectangle in Fig. 1a (more explanation in Supplementary Figure S1).

### Spin–orbit torque induced magnetization switching

Measurement of SOT induced magnetization switching includes three sequences: initialization, SOT writing by pulse current, and reading from Hall resistance measurement. Firstly, sample is under strong enough + z-axis (− z-axis) direction external field to saturate the magnetization in up (down) direction as initial state. After initialized, the current pulse is injected with pulse amplitude (*I*_*p*_) from − 25 to 25 mA (25 to − 25 mA) with pulse width of 10 ms. During the current pulse injection in x-axis direction in-plane magnetic field is applied to ensure deterministic switching. To detect SOT induced switching, the Hall voltage (*V*_*H*_) is measured in the middle of each pulse injection using 100 μA direct current (DC). Here, we should mention that the Hall resistance (*R*_*H*_) is unit value calculated from measured *V*_*H*_ dividing with magnitude of reading current. The *R*_*H*_ results as a function of *I*_*p*_ with the previous procedure shows typical hysteresis loops, as shown in Fig. 1b–c, and it indicates SOT induced magnetization switching in PMA system. Here, the shown hysteresis loops in Fig. 1b are in case for dose amount of 30 ions/nm^2^. It is well known that higher in-plane field make switching more easily, but we observe that helium ion irradiation also reduces the switching current. The SOT driven magnetization switching hysteresis loops for various doses with a fixed 2.2 kOe external in-plane field is shown in Fig. 1c. In Fig. 1d, the switching currents from magnetization switching loops at each dose and external magnetic field are depicted. In Fig. 1d, we found two features. Firstly, in small field region (0.7 kOe), rapid increase of switching current appears. We expect that this increase is caused by the nucleation of multi-domain states during SOT induced magnetization switching process (see the Supplementary Figure S2a for more details). Because of the multi-domains under small field, it is hard to compare the switching current in higher field region directly so that we will not pay attention much. The second feature is main finding of this work in larger field regions (≥ 1.2 kOe). We found that the switching current is reduced by increasing dose amounts. And at same dose amount, the switching current have linear relation with the external in-plane field strength under the sufficiently smaller in-plane field comparing 1st order effective anisotropy field, which is well-known behavior following ${J}_{C}=\frac{2e}{\hslash }\frac{{M}_{s}{t}_{F}}{{\theta }_{SH}}\left(\frac{{H}_{K,eff}}{2}-\frac{{H}_{x}}{\sqrt{2}}\right)$^28^. And here, e is charge of electron, *ℏ* is Planck constant, *M*_*s*_ is saturation magnetization, *t*_*F*_ is thickness of FM layer, *H*_*K,eff*_ is the 1st order anisotropy field and *H*_*x*_ is in-plane external magnetic field parallel with current. In order to get better insight of the helium irradiations effect, we show the switching current reduction ratio at each dose compared with the pristine sample ($\left|\left({I}_{P.crit}-{I}_{P,crit}^{Dose 0}\right)/{I}_{P,crit}^{Dose 0}\right|\times 100 \%$) as in Fig. 1e. Result shows that the switching current reduction has increasing tendency with dose amount for in-plane external field. The exceptional dependence for small field (0.7 kOe) probably ascribe to the formation of multi-domain state as seen in Supplementary Figure S2b. The reduction ratio appears largely at external field of 3.1 kOe about 14.2%, 25.5%, and 30.3% at dose of 10, 20, and 30 ions/nm^2^, respectively. Here, the possible physical origins of the switching current reduction can be the enhanced *θ*_*SH*_ of Pt layers, and/or it can be the decrease of the effective anisotropy field of FM layer. We will discuss more details later.

### Determine the 1st and 2nd order uniaxial anisotropy fields

**Figure 2:**
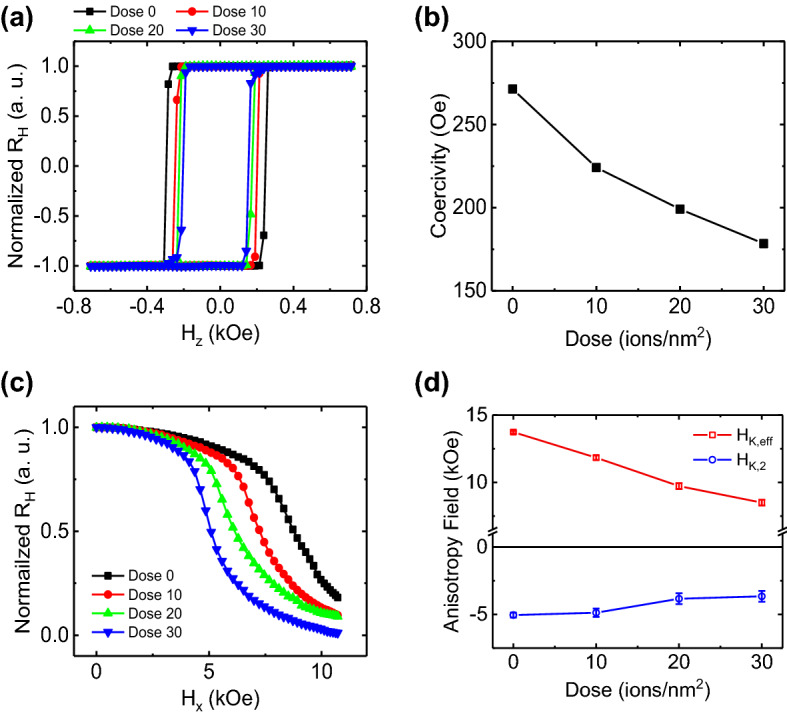
AHE measurement results for Pt(5)/Co(0.8)/MgO(2 nm) samples ay various helium ion irradiation doses amount. (**a**) Normalized AHE loops with the external magnetic field perpendicular (*H*_*z*_) for various doses. (**b**) The coercivities from the AHE loops as a function of doses. (**c**) Normalized AHE signals of various doses with the in-plane field (*H*_*x*_) for GST method. Here, the solid lines are trending lines. (**d**) The 1st and 2nd anisotropy fields (*H*_*K,eff*_, *H*_*K,2*_) extracted by the GST method are plotted for various doses. We measured all AHE with the reading current of 100 μA for various doses of 0, 10, 20, and 30 ions/nm^2^. Current flow along x-axis and Hall voltage is measured in y-axis. Here, the error bars in (**b**) are smaller than symbol size.

To understand the more details of switching behavior, the effects of helium ion irradiation on the magnetic anisotropy fields are investigated. We conducted Anomalous Hall effect (AHE) measurement by swapping the external magnetic field in z-axis direction to obtained normalized Hall resistance (*R*_*H*_*(H*_*ext*_*)/R*_*H*_*(H*_*ext*_ = *0 Oe)*) hysteresis loops, because the AHE signal is proportional to the z-component of magnetization. The normalized AHE hysteresis loops in Fig. 2a shows strong enough PMA for all samples. And each coercivity is decreasing (~ 34%) from 271 to 178 Oe as shown in Fig. 2b by increasing dose amounts. In order to obtain the 1st and 2nd order anisotropy fields (*H*_*K,eff*_, *H*_*K,2*_) by using generalized Sucksmith-Thompson (GST) method (see Supplementary Figure S3), we measured normalized AHE by applying in-plane field (*H*_*x*_) along the current direction as seen in Fig. 2c. Here, it must be mentioned that the obtained 1st order anisotropy fields are the effective anisotropy including demagnetization effect, not pure anisotropy field in GST method. The *H*_*K,eff*_ and *H*_*K,2*_are shown in Fig. 2d as a function of dose. By increasing dose amount from 0 to 30 ions/nm^2^, *H*_*K,eff*_ and *H*_*K,2*_decrease 38.2%, and 27.5%, respectively. We speculate that decrease is mainly caused by interface modulation from helium ion irradiation process, since the surface anisotropy energy is very sensitive on the quality of the interface between HM and FM layers. Although it is hard to classify and/or probe the effect of the structural modulation caused by the helium ion irradiation, we can claim that he anisotropic field as well as the coercivity field can be reduced by the helium ion irradiation. The magnitude of *H*_*K,2*_ is only less than half (40.1%) compared with the *H*_*K,eff*_, however, the SOT analysis without consideration of *H*_*K,2*_ may lead incorrect results^29^.

### Spin–orbit torque induced effective fields by harmonic Hall measurement

**Figure 3:**
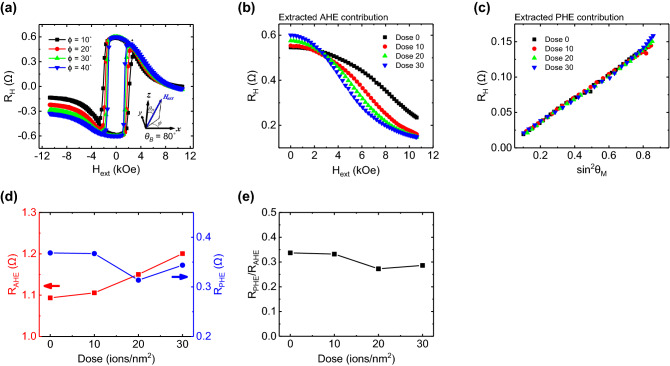
AHE and PHE resistances measured for various doses. (**a**) Hall resistance loops of dose amount of 30 ions/nm^2^ in *ϕ* = 10°–40°. The oblique magnetic field (*θ*_*B*_ = 80°) is applied to obtained mixed Hall resistances signals. (**b**) AHE contribution and (**c**) *R*_*H*_ as a function of sin^2^ *θ*_*M*_ to extract the PHE contribution from the slopes of those plots. The measurements results are obtained for various doses at *ϕ* = 40° at *θ*_*B*_ = 80°. (**d**) Resulting R_AHE_ and R_PHE_ values and (e) its ratio, R_PHE_/R_AHE_, at each dose. Here, the error bars in (**d**) and (**e**) are smaller than symbol size.

Not only the anisotropy characteristics, but also an important parameter in SOT induced magnetization reversal is *θ*_*SH*_. The harmonic Hall signal analysis is frequently used method for calculating *θ*_*SH*_ as well as extracting SOT driven effective fields^29,30^. It is well known that the SOT has two contributions acting on different directions, so called field-like torque (FLT, *ΔH*_*FL*_) in transverse direction and damping-like torque (DLT, *ΔH*_*DL*_) effective field in longitudinal direction, consideration of AHE and planar Hall effect (PHE) resistances are necessary for obtaining correct results. In Fig. 3a, the measured Hall resistance loops are shown at *ϕ* = 10°–40° with fixed *θ*_*B*_ = 80° for dose amount of 30 ions/nm^2^ sample. Since the AHE and PHE contribute to the measured Hall signal as following the equation^30^,

1 $${V}_{H}={I}_{0}{R}_{0}=\frac{{I}_{0}{R}_{AHE}}{2}\mathrm{cos}{\theta }_{M}+\frac{{I}_{0}{R}_{PHE}}{2}{\mathrm{sin}}^{2}{\theta }_{M}\mathrm{sin}2\phi$$

The clear asymmetries are observed for the Hall loops in Fig. 3a in the large field. The asymmetry also increases because of the larger PHE contribution for large *ϕ*. The AHE and PHE contribution can be extracted using Eq. (1) and Fig. 3b and c show the extracted contributions at the angle of *θ*_*B*_ = 80° and *ϕ* = 40° at each dose amount. Details of extracting method for AHE and PHE resistances is explained in Supplementary Figure S4. From those measurement analyses, the calculated AHE and PHE resistances are shown in Fig. 3d together. R_AHE_ changed from 1.09 to 1.20 Ω (9.8%) with increasing dose amounts, while R_PHE_ varied within the range of 0.37 to 0.34 Ω. Since the ratio of *R* = *R*_*PHE*_*/R*_*AHE*_ has an important role in analysis of the harmonic Hall measurement result, we calculated the ratio and it changes from 0.34 to 0.29 at dose of 0 and 30 ions/nm^2^, as depicted in Fig. 3e. It seems like that the variation of R by the degree of helium ion irradiation is not having significant changes comparing to other physical quantities, but it has an important role in harmonic Hall signal analysis.

**Figure 4:**
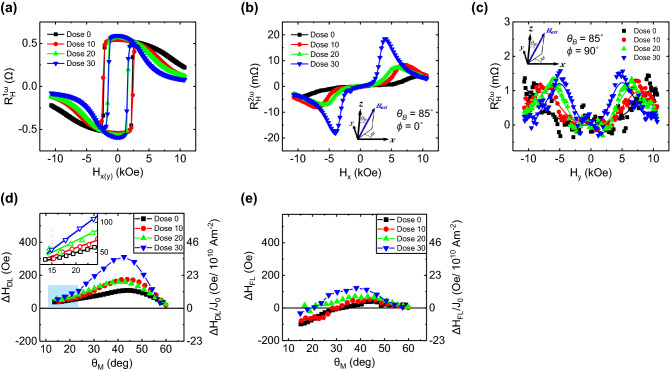
1st and 2nd harmonics measurement results for SOT induced effective field. AC is injected into Hall bar structure with amplitude of 5.5 mA and frequency of 401 Hz for the harmonics measurements. External field is applied with *θ*_*B*_ = 85° and with direction parallel to x-axis (*ϕ* = 0°) and y-axis (*ϕ* = 90°) for measuring *ΔH*_*DL*_ and *ΔH*_*FL*_ effective fields, respectively. (**a**) 1st harmonics (${R}_{H}^{1\omega }$) (**b**) 2nd harmonic (${R}_{H}^{2\omega }$) for *ϕ* = 0° and (**c**) ${R}_{H}^{2\omega }$ for *ϕ* = 90° at various doses. Each solid line is trending line. (**d**) Extracted *ΔH*_*DL*_ and (**e**) Δ*H*_*FL*_ as function of *θ*_*M*_ considering *H*_*K,eff*_ and *H*_*K,2*_.

To obtain the *θ*_*SH*_ or SOT induced effective fields, we measure harmonic Hall with alternating current (AC) of 5.5 mA peak amplitude and 401 Hz frequency (*I*_*AC*_ = *I*_*0*_ sin2π*ft*). Because the harmonic Hall measurement is influenced by the Joule heating effect caused by current flow, we follow the four-direction method for eliminating some thermoelectric artifacts^31^. The 1st and 2nd harmonic Hall loop is measured swapping magnetic field with fixed *θ*_*B*_ = 85° and *ϕ* = 0° for *ΔH*_*DL*_ and *ϕ* = 90° for *ΔH*_*FL*_ measurements. Each Hall loop result is shown in Fig. 4a–c, respectively. Here, the 1st and 2nd harmonics are measured simultaneously with two lock-in amplifiers (LIA) at each *ϕ* and dose amounts. Harmonic Hall voltage signal under AC follows the equation,

2 $${V}_{H}={I}_{o}{R}_{H}={V}^{1\omega }\mathrm{sin}\left(\omega t\right)-{V}^{2\omega }\mathrm{cos}(2\omega t)$$

Although 1st voltage has almost same signal at each *ϕ*, 2nd voltage has completely different signals as shown Fig. 4b and Fig. 4c. These results are come from the different contribution between DLT and FLT. The 2nd order harmonic Hall voltage at each *ϕ* = 0° and 90° with consideration of 2nd order PMA energy follow the expression^29^,

3 $${V}_{x}^{1\omega }={V}_{y}^{1\omega }={V}_{AHE}\mathrm{cos}{\theta }_{M}$$

4 $${V}_{x}^{2\omega }=\frac{{V}_{AHE}}{2} \left({A}_{1}\Delta {H}_{DL}-{B}_{1}\Delta {H}_{FL}\right)$$

5 $${V}_{y}^{2\omega }=\frac{{V}_{AHE}\mathrm{cos}{\theta }_{M}}{2} ({B}_{1}\Delta {H}_{DL}-{A}_{1}\Delta {H}_{FL})$$

6 $${A}_{1}\equiv \frac{\mathrm{sin}{\theta }_{M}}{{H}_{K,eff}\mathrm{cos}2{\theta }_{M}-{H}_{K,2}\mathrm{sin}{\theta }_{M}\mathrm{sin}3{\theta }_{M}+{H}_{ext}\mathrm{cos}\left({\theta }_{M}-{\theta }_{H}\right)}$$

7 $${B}_{1}\equiv \frac{R{\mathrm{sin}}^{2}{\theta }_{M}}{{H}_{ext}\mathrm{sin}{\theta }_{H}}$$

Following the Eqs. (2)–(7), we can rewrite DLT and FLT effective fields (*ΔH*_*DL*_, *ΔH*_*FL*_) as function of *θ*_*M*_ from the measured harmonic Hall voltages as shown in Fig. 4d and e, respectively. Here, *θ*_*M*_ can be calculated using experimentally obtained the 1st order harmonic Hall signal at each dose with Eq. (3). The results show the different dose dependences on *ΔH*_*DL*_ and *ΔH*_*FL*_ with *θ*_*M*_. When the near of *θ*_*M*_ = 15°, corresponding magnetization angle at external magnetic field of 3.1 kOe in 0 ions/nm^2^, *ΔH*_*DL*_ has small increasing tendency as shown in the inset in Fig. 4d but *ΔH*_*FL*_ has decreasing tendency at its magnitude according to dose amount. However, when *θ*_*M*_ > 15°, both effective fields show great increase and complex behavior having a maximum peak point at *θ*_*M*_ of range from 40° to 45°. According to simple macro-spin SOT model^29^, there is no magnetization direction dependence on both effective SOT fields. However, there are much experimental evidences of the magnetization direction dependence on the effective SOT fields^30–32^. The higher order term of SOT can be one of the possible origins of complex angular dependence. According to Ref.^30^, the high order term of SOT is non-negligible and may cause complex angular dependence. In addition, if helium ion irradiation modulates the higher-order term of the SOT just similar as the higher-order term of the PMA, the change in angular dependence can be estimated as a phenomenon caused by the helium ion irradiation. Furthermore, another possible approach explaining such magnetization direction dependent effective SOT fields is from the framework of distorted Fermi surface^33^. The *θ*_*M*_ dependent effective fields in Fig. 4d, e are rather complicated angular dependence compared with the theoretical results are based on the free-electron like model Hamiltonian with exchange coupling and Rashba effect. The experimental results reflect realistic band structures so that the more complex angular dependent explanation is acceptable. It is hard to analysis the exact origins separately. However, it is also true that the varying angular dependent effective fields by degree of ion irradiation has been experimentally observed as seen in Fig. 4d, e. And it is worth to note that if *H*_*K,2*_ is not considered in the calculation, the result has quite different tendency with Fig. 4d, e, suggesting the critical role of the 2nd order anisotropy in precise analysis of harmonic Hall measurement in all range of *θ*_*M*_ (see the Supplementary Figure S5).

### Temperature dependent resistivity measurement on single platinum layers

**Figure 5:**
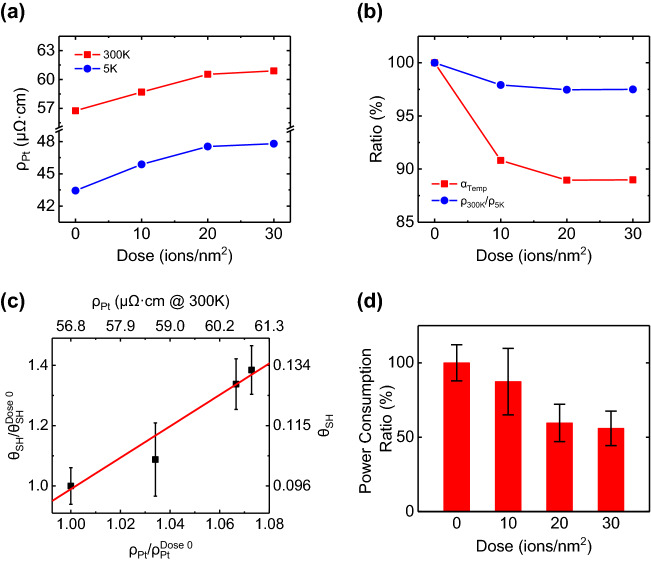
Resistivity changes of Platinum single layer for various doses and its comparison with SHA. (**a**) Measured Platinum single layer resistivity at 300 K and 5 K as function of dose amounts. (**b**) The ratios of temperature coefficient *α*_*Temp*_ and *RRR* comparing to the pristine sample values (**c**) Comparison of *θ*_*SH*_ and resistivities at 300 K for various doses. Red solid line is linear fitted line of *θ*_*SH*_ with platinum resistivity. (**d**) Calculated power consumption ratio at each dose comparing pristine sample. Here, the error bars in (**a**) and (**b**) are smaller than symbol size.

Because *θ*_*SH*_ is one of the most important material parameters for SOT based devices, understanding the correlation between ion irradiation induced *θ*_*SH*_ variation and HM layer state is important. In order to reveal the effect of the helium ion irradiation on HM layer only, we irradiated the helium ion on single Pt layer with thickness of 5 nm as same conditions introduced in sample fabrication description. We used the 4-probe measurement technique for measuring resistance with temperature range of 5 K to 225 K and calculated resistivity using sample geometry information with measured resistance. Here, the resistivity curve and the method of calculating resistivity at 300 K are explained in Supplementary Figure S6a. The resistivity of Pt (*ρ*_*Pt*_) at 300 K and 5 K is shown in Fig. 5a and we can observe the increasing resistivity according to dose amount, 43.4–47.8 μΩ cm (109%) in 5 K and 56.8–60.9 μΩ cm (107%) in 300 K comparing 0 ions/nm^2^ and 30 ions/nm^2^. Figure 5b displays the changed ratio of temperature coefficient (*α*_*Temp*_), following *ρ* = *ρ*_*0*_* (1* + *α*_*Temp*_* ∙ (T* − *T*_*0*_*)* at linear resistivity increasing region (*T* > 50 K), and residual-resistivity ratio (*RRR*), comparison of resistivities between 300 and 5 K in here. We can find the decreasing tendency of *α*_*Temp*_ and *RRR* both, it can be interpreted as increased influence of impurity at higher dose. Because the collision time is inversely proportional to the impurity density, decrease of *α*_*Temp*_ and *RRR* value imply that the helium ion irradiation makes extra scattering sources by structural distortion in Pt layer. The Fig. 5c shows the resistivity dependence of *θ*_*SH*_, and it can be calculated with *ΔH*_*DL*_ at each dose value using following equation^34^,

8 $${\theta }_{SH}=\frac{2\mathrm{e}}{\mathrm{\hslash }}\frac{{M}_{s}{t}_{F}{A}_{HM}}{{I}_{0}}\Delta {H}_{DL}$$

Here, A_HM_ is the cross section area of flowing current into HM. We assume that the influence of irradiation on *M*_*s*_, *t*_*F*_, and *A*_*HM*_ is small enough to ignore, because the irradiated dose amount is scarce to cause interlayer deformation^19,20,27^. So, we calculated the *θ*_*SH*_ with *M*_*s*_ = 1100 kA m^−1^, *t*_*F*_ = 0.8 nm, *A*_*HM*_ = 10 μm × 5 nm and *ΔH*_*DL*_ when *θ*_*M*_ = 15° at each dose amount using Eq. (8). Error bar can be calculated as the averaged of values of the front and rear data starting from *θ*_*M*_ = 15°. It is found that *θ*_*SH*_ has linear relation with increased resistivity by helium ion irradiations, as well reported^11,35,36^. The *θ*_*SH*_ increases 0.096 to 0.132 with resistivity growth from 56.8 to 60.9 μΩ cm in 300 K, about 5–6 times greater than resistivity of bulk Pt (10.6 μΩ cm in 20 ℃) in literature^37^. This result suggests that helium ion irradiation process makes extra scattering sources, and they raise the resistivity of HM layer. And the extra scattering sources cause improvement of *θ*_*SH*_, resulting in more effective switching of the magnetization by SOT. Although the energy efficiency in view of operating the device is slightly worse due to the ion irradiation induced resistance increasement, the improved *θ*_*SH*_ ratio is ~ 4 times greater compared with resistivity increasement ratio. In terms of power (*P* = *R*_*sample*_*I*^*2*^) consumption, the change in resistance and SHA has an inverse relationship. As a result, only 87.4%, 59.6%, and 56.0% of power consumption is expected at 10, 20, and 30 ions/nm^2^, respectively. (See Fig. 5d) Here, the *R*_*sample*_ and *I* are normalized resistance of HM and normalized current by *θ*_*SH*_ at each dose. Therefore, it means that helium ion irradiation enables more efficient data writing in terms of energy consumption. And it is worth mentioning that the critical switching current equation shown in Ref.^28^ does not match with our actual experimental value except only linear relationship with in-plane field. We expect because the formula is based on the macro spin model as like well-known Brown paradox^38^. Furthermore, there are reports that it does not match the actual value in the micron scale sample^39,40^. That’s why we obtained *θ*_*SH*_ from the spin–orbit torque effective field measurement (see Fig. 5c), not from the switching current density. Nevertheless, it is clear that the helium ion irradiation leads to a decrease in the *H*_*K,eff*_, an increase in the *θ*_*SH*_, and the more efficient the SOT induced magnetization switching.

In summary, we observe that helium ion irradiation can properly reduce SOT induced switching current in Pt(5)/Co(0.8)/MgO(2) structure. The reduction appears 14.2%, 25.5%, and 30.3% at dose of 10, 20, and 30 ions/nm^2^ comparing with the pristine sample under the in-plane external magnetic field of 3.1 kOe. For understanding of physical reasons of decreasing tendency of the switching current, we considered two main possible origins of reduction, *H*_*K,eff*_ and *θ*_*SH*_. From AHE measurement and GST method, we can extract *H*_*K,eff*_ and it decreases from 13.7 to 8.5 kOe (38.2%) comparing dose 0 and 30 ions/nm^2^. Not only *H*_*K,eff*_, *θ*_*SH*_ also increase from 0.096 to 0.132 (27.2%). Furthermore, it is revealed that improvement of *θ*_*SH*_ is consequence of increase of Pt resistivity by ion irradiation process. Although the power consumption is slightly worse due to the increase of the resistance, the decreased critical current caused by the improved *θ*_*SH*_ has a greater impact in power consumption. As a result, the ratio of power requiring for operation of device is calculated to consume only about 56.0% for switching at 30 ions/nm^2^ compared to pristine sample, and this successful analysis on helium ion irradiation induced modulation of SOT effect can be expected to improve efficiency of SOT based spintronic devices engineering.

## Methods

### Thin film preparation and fabrication process

The sample preparation process includes three steps, firstly Hall bar photolithography and deposition, secondly electrode fabrication, and helium ion irradiation at last. This section will only explain up to step 2, and step 3 will be explained in later paragraph (See the “Helium ion microscope” in Methods section). The sample is fabricated with lift-off process using photolithography and magnetron sputtering system. Normal metal layer and oxide layer is deposited using DC and AC power with stack of Pt(5)/Co(0.8)/MgO(2) on single surface polished Si substrate having 300 nm thickness SiO_2_ oxidated surface. The patterned Hall bar geometry consists of the current line of 10 μm width and 40 μm length and voltage line of 3 μm width 16 μm length. This geometry represses the offset error caused by sample shape. After deposition and patterning of sample, we deposit the electrode with stack of Ta(5)/Cu(50) for electric measurement.

### Helium ion microscope

Helium ion microscope (HIM, Carl Zeiss/ORION NanoFab), using high energy ionized helium, is one of the brand-new microscopic techniques. Using HIM, we can obtain more detailed image in nano-scale structure compared with Gallium ion or electron based microscope owing to its penetration characteristic of high energy helium ion^20,27^. But for utilizing HIM, we must make trimer, as the state of leaving only three atoms at the end of tip, stability and duration problems are remained. Even in such problems, the high penetration characteristic and low diffraction limitation of HIM come to be a big attraction. Because the HIM have low convergence angle, long best focus length and high penetration depth, more than few tens of nanometer scale, it also can be used as precise atomic structure destruction method. We expose the helium ion with normal direction of sample plane with dose value of 0–30 ions/nm^2^ at energy of 30 keV and current of 5.4 ~ 5.5 pA. The exposure process can be conducted from few hundred micron to few tens of nanometer size, and we conducted with area of 20 μm × 20 μm. The HIM is in the Central Core Research Facility center in DGIST, Korea.

### Transport measurement system with DC and AC current

For measuring SOT induced magnetization switching, we use the DC & AC source (Keithley, 6221) and nano-voltmeter (Keithley, 2182A). Each equipment is connected to sample using customized PCB in the 2-axis rotational holder. The direction of magnetic field can be controlled by rotating the holder in prefer direction. For measuring SOT induced magnetization switching, we use the current source (6221) for injecting pulse current and nano-voltmeter (2182A) for measuring *V*_*H*_. The magnetization is initialized to + z direction (or − z direction) using magnetic field with larger than coercivity of sample. The SOT induced switching is conducted using 10 ms width pulse and each pulse is injected to sample having pulse amplitude from − 25 to 25 mA with 1 mA gap Here, the time gap between pulse to pulse is set as 1 s. During pulse to pulse time gap, the small amplitude DC current (0.1 mA) is injected for observing the degree of switching. All current injection is conducted under constant in-plane direction (aligned to x-axis) external magnetic field.

Comparing SOT induced magnetization switching measurement, the harmonic Hall measurement use two lock-in amplifiers (LIA, Stanford Research, SR830) for detecting AC based Hall signals with same current source (6221). One LIA detects the 1st harmonic signal, and the other detects the 2nd signal. All LIAs are connected to sample with same voltage line, so the 1st and 2nd harmonic signal is measured simultaneously. The used AC has 5.5 mA peak amplitude and 401 Hz frequency. The 1st and 2nd harmonic Hall loop is measured swapping magnetic field from − 1 T to + 1 T with sweep rate of 500 ms and polar angle of magnetic field is fixed to 85° for avoiding multi-domain nucleation during harmonic Hall measurement. The azimuthal angle is measured at two cases of *ϕ* = 0° and *ϕ* = 90° for SOT induced longitudinal and transverse contribution, respectively. Because the thermoelectric artifacts can be influence on harmonic Hall signal, the measured data is post-processed following Ref.^31^.

For temperature dependent measurement, we use the cryostat with built-in PCB connector. The resistance is measured using 4-probe measurement by current source (6221) and nanovoltmeter (2182A). The fabricated sample geometry is current line of 10 μm width × 40 μm length, and the gap distance between voltage detection positions is 12 μm. sample atmosphere temperature can be controlled using chamber heater and helium compressor system. The temperature increases from 5 to 275 K with gap of 0.25 K, and between each temperature, resting time is 5 min for stabilizing atmosphere. The DC current is injected to sample during whole measurement with amplitude of 0.1 mA.

## Supplementary Information

Supplementary Information.
