# Supplementary material for: Improved spin–orbit torque induced magnetization switching efficiency by helium ion irradiation
Source: Sci Rep. 2022 Mar 2;12:3465. doi: 10.1038/s41598-022-06960-8 (PMC8891290; doi:10.1038/s41598-022-06960-8)
Supplement: Supplementary file 1 — Supplementary Information. [file 41598_2022_6960_MOESM1_ESM.pdf]

Improved Spin-Orbit Torque Induced Magnetization Switching Efficiency by Helium Ion Irradiation

Suhyeok An[1], Eunchong Baek[1], Jin-A Kim[1], Ki-Seung Lee[2 *] and Chun-Yeol You[1,*]

[1] *Department of Emerging Materials Science, DGIST, Daegu 42988, Korea*

[2] *Emerging Materials Science Research Center, DGIST, Daegu 42988, Korea*

Contents

Supplementary Note 1: Local Helium ion irradiation and anomalous Hall signal differences

Supplementary Note 2: Multi-domain states in spin-orbit torque induced switching with small external in-plane magnetic field

Supplementary Note 3: Generalized Sucksmith-Thompson method

Supplementary Note 4: The method of Distinguish the anomalous and planar Hall effect contributions

Supplementary Note 5: Contribution of Harmonic Hall result according to existence of $H_{K,2}$ consideration in spin-orbit torque analysis

Supplementary Note 6: ρ_{xx} and ρ_{yx} of Platinum single layer with He^+ ion irradiations

Supplementary Note 1: Local Helium ion irradiation and anomalous Hall signal differences

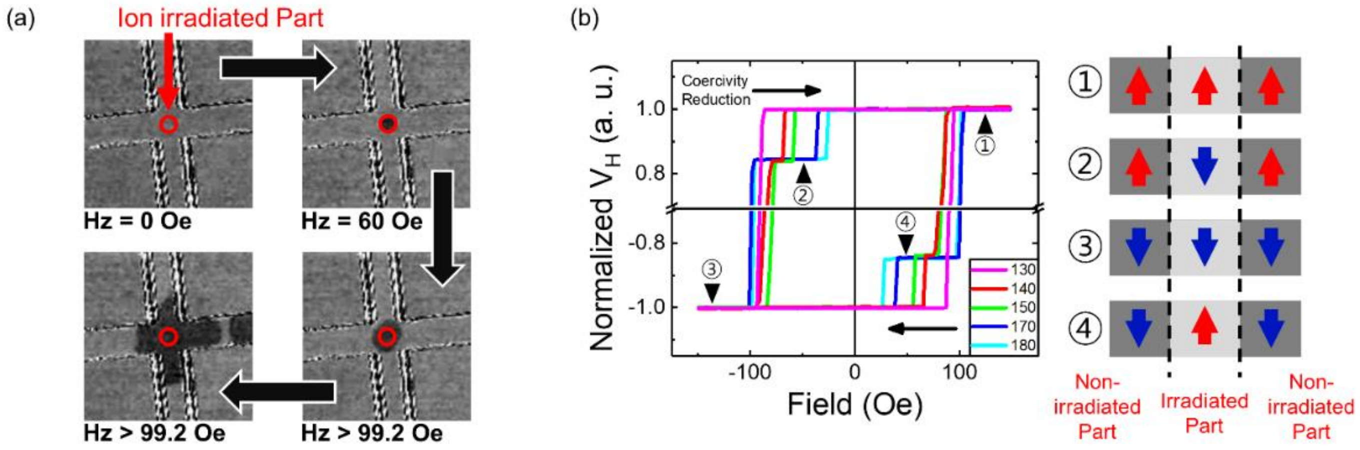

Figure S1. Local switching of magnetization by perpendicular direction magnetic field in Pt(5)/Co(0.6)/Pt(5) structure. (a) Magneto-optical Kerr microscope result at various external field with dose amount of 150 ions/nm^2. The He$^+$ ion irradiated only local area, denoted by red circles. External field increases gradually from 0 to 99.2 Oe in z-axis and stop when the domain starts to spread. (b) Measured anomalous Hall effect result at various dose amounts. Here, the the dark gray indicates non-irradiated part on Hall cross and light gray is irradiated part. Each number shows magnetization configuration at that state in hysteresis loop.

For understanding local change of magnetic properties, we demonstrate effect of local irradiation. The He$^+$ ion is irradiated with 25 μm diameter dot shape on the center of Hall cross with 50 μm width in Pt(5)/Co(0.6)/Pt(5) structure as denoted by red circles in Fig. S1a. Fig. S1a shows magneto-optical Kerr effect (MOKE) images in various external magnetic field in dose amount of 150 ions/nm^2. It is observed that the magnetization of irradiated part switches firstly in smaller external field than coercivity (H_c), and then the domain is spreading at H_c (99.2 Oe). This local switching means that the H_c is reduced by ion irradiation process. The MOKE images clearly show the magnetic property can be engineered only irradiated area.

To extract more detailed information, we measure the anomalous Hall effect (AHE) from swapping external magnetic field from -250 to 250 Oe in z-axis direction with 1 mA DC reading current. The resulting AHE hysteresis loop is shown in Fig. S1b at dose amount from 130 to 180 ions/nm^2. We can easily observe the reduction of H_c in irradiated part only by steps in the hysteresis loops. The local switching is described using schematic diagram on right of AHE data. Each number indicates magnetization state of Hall cross at each case on AHE result. Here, the light gray part is corresponding to irradiated part and dark gray is non-irradiated part. Reduction appears 99.2 Oe to 30.5 Oe (69.3%) comparing the pristine and 190 ions/nm^2 dose sample. The

step height in AHE voltage differences is about 15% at all doses. It is expected that the area ratio between irradiated part and Hall cross ($A_{Irradiation}/A_{Hall\ Cross}$) has 19.6%, and because influenced area by Hall measurement is slightly wider than $A_{Hall\ Cross}$, actual area ratio can be smaller. Furthermore, AHE voltage differences has dependence on magnetization, almost similar AHE voltage changes by ion irradiation with different dose amounts indicate that the irradiation process does not give big influence on the saturation magnetization. Since these changed properties are observed at the same time by AHE measurement, for obtaining only changed properties, we need to cover He^+ ion irradiation area wider than Hall cross area for avoiding mix-up of signals between irradiation and non-irradiation part.

Supplementary Note 2: Multi-domain states in spin-orbit torque induced switching with small external in-plane magnetic field

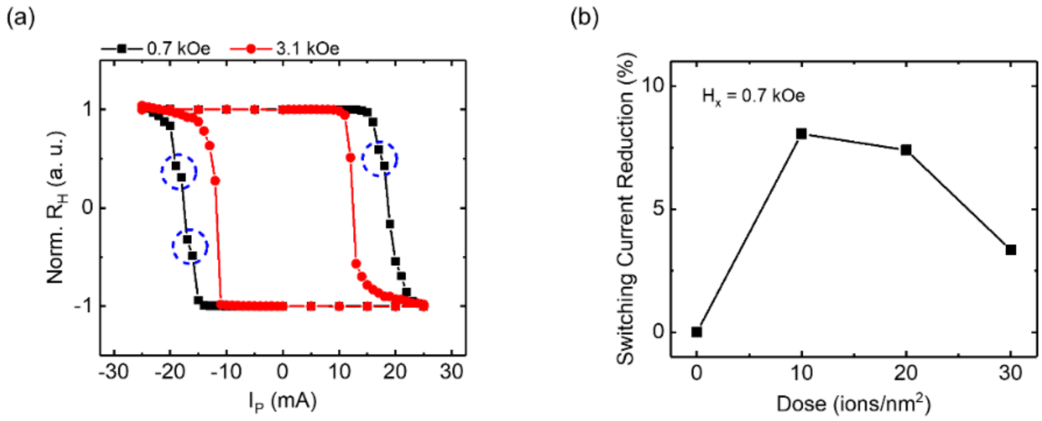

Figure S2. (a) SOT induced hysteresis loop comparison between 0.7 and 3.1 kOe at dose amount of 20 ions/nm2. The blue circles in 0.7 kOe hysteresis loop indicate multi-domain nucleation induced middle points during magnetization switching. (b) Calculated switching current reduction ratio ($\left|\left(I_{P.crit} - I_{P,crit}^{Dose\ 0}\right)/I_{P,crit}^{Dose\ 0}\right| \times 100\ \%$) at in-plane external field of 0.7 kOe.

In the magnetization switching process with the magnetic field, the experimentally observed switching (coercivity) field is much smaller than the anisotropy field, which is so called Brown paradox [1]. According to the Brown paradox, the switching at the anisotropy field is only valid in single domain model, and real switching field can be gratefully reduced by formation of multi-domain state and domain wall motion. Such discrepancy is also found in the SOT induced switching in our study.

The macro-spin model using the single domain model and the actual observed values are quite different, which is thought to be due to the generation of multi-domain states during the switching process. We observed that different domain nucleation states appear according to the magnitude of in-plane direction external magnetic field. In Fig. S2a, initially abrupt switching occurs, but slow switching is dominant at the end of switching for 3.1 kOe loop. However, multi-domain effect is more dominant in the low field region. In the case of a small field (0.7 kOe), a middle-step at blue circles in Fig. S2a is observed, and it is assumed that switching occurs by formation of the bubble domains by the reason that sufficient SOT is not applied for forming the domains. However, as the field increases, the middle-step disappears, and it is assumed that the switching proceeds to single-domain nucleation due to sufficient SOT. Therefore, we considered that the two domain nucleation states are in different causes, and thought that it would be difficult to directly compare the small field region with the higher field region. In fact, as shown in Fig S2b, the switching current reduction ratio at 0.7 kOe showed a tendency to decrease according to the dose amount (middle steps are observed) while increasing tendency is observed in another external field region (no middle steps).

Supplementary Note 3: Generalized Sucksmith-Thompson method

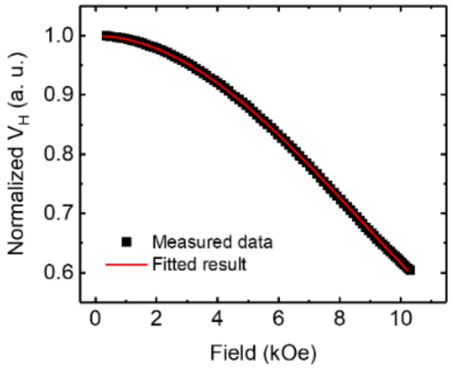

Figure S3 Normalized Hall resistance and its fitted result. The black dot is measured and normalized data with $(R_H(H_{ext})/R_H(H = 0 \text{ Oe}))$. And the red line is fitted result using GST method

Because the modulated area by ion irradiation in our system is micron scale size, it is hard to directly measure or configure changed magnetic anisotropy energy by irradiations. So, we follow the generalized Sucksmith-Thompson (GST) method [2,3] to extract the 1st and 2nd order perpendicular magnetic anisotropy

(PMA) field. We used AHE measurement to apply GST method, where AHE is proportional to the z-component of the magnetization. And the more than whole Hall cross area are irradiated to guarantee to exclude the un-irradiated area signal as shown in Fig. 1a. The GST method follow the equations,

$$H_K^{eff} + H_{K,2}(1 - m^2) = \alpha H_{ext} \qquad (1)$$

$$\alpha = \frac{m \sin\theta_H - \sqrt{1-m^2}\cos\theta_H}{m\sqrt{1-m^2}} \qquad (2)$$

Here, H_K^{eff} is the first order PMA effective field, $H_{K,2}$ is the second order PMA field, $m = \cos\theta_M$, θ_M is the polar angle of magnetization and θ_H is the polar angle of external magnetic field . FIG. S2 shows the fitted result using equation (1) and (2). The result in Fig.1 is shown with measured data under direction of ϕ = 0° and θ_H= 80° with the pristine sample. As seen in Fig. S2, although the applied field is much smaller than PMA field of sample, precise determination H_K^{eff} and $H_{K,2}$ is possible. The obtained H_K^{eff} and $H_{K,2}$ are depicted in Fig. 2d as a function of dose amount.

Supplementary Note 4: The method of Distinguish the anomalous and planar Hall effect contributions

In Hall measurements, if the applied magnetic field is out-of-plane, only anomalous Hall effect (AHE) is detected. And only the planar Hall effect (PHE) is considered for the in-plane direction in PMA. However, with general direction field as like harmonic measurements, we have always mixed signals of AHE and PHE. Therefore, careful analysis is required to distinguish both contributions in the harmonic measurements. In normally, the AHE measurement only needs small external field near coercivity, but PHE contribution change are detected with azimuthal angle (ϕ) dependence inside external in-plane direction magnetic field exceeding PMA effective anisotropy field. However, in our system, the PMA field has high enough, few thousand to ten thousand Oersted scale, we follow the separating method between AHE and PHE contribution using asymmetrically measured Hall voltage [4] to extract the ion irradiation induced AHE and PHE contribution change within field magnitude of 1 T. Fig. S3a and Fig. S3b show the separated plot, measurement of +B to -B and -B to +B, in original loop and only -B to +B part reversed loop in θ_B = 80° and ϕ = 40°. Fig. S3b clearly show asymmetricity by measurement direction caused by PHE contribution. Adding and subtracting

each measurement results, we can calculate AHE and PHE contributions as shown in Fig. S3c and Fig. S3d .

From the AHE contribution, we can calculate AHE resistance and θ_M following,

$$R_{H,AHE}(H) = R_{AHE}\cos\theta_M \quad (3)$$

$$\theta_M = \cos^{-1}\frac{R_{H,AHE}(H)}{R_{AHE}} \quad (4)$$

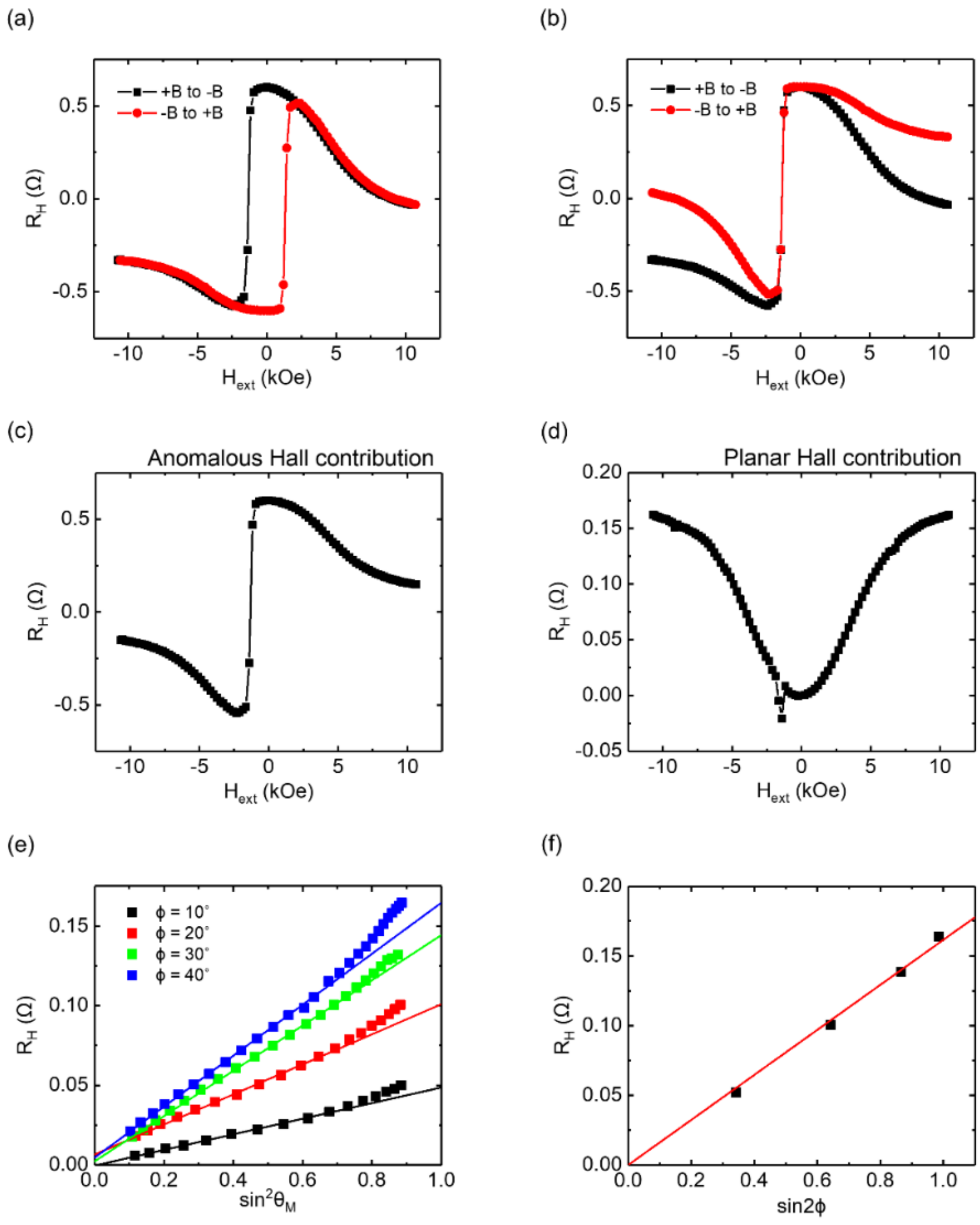

Figure S4. AHE and PHE contribution extracting method using Hall resistance measurement. (a) Hall resistance result measured at magnetic field direction of θ_B of 80° and ϕ of 40°. (b) Reoriented data flipping measurement data of -B to +B. (c) Extracted AHE contribution and (d) PHE contribution. (e) rewritten data of PHE contribution with function of $\sin^2\theta_M$, and each line means linear fitting results. (f) Slope data at each $\sin 2\phi$ is in black square dot and its linear fitted line in red line.

Using equation (4), magnetic field can directly match with θ_M and we can replot the PHE contribution as function of $\sin^2\theta_M$. Because the PHE contribution follows the equation,

$$R_{H,PHE}(H) = R_{PHE}\sin^2\theta_M \sin 2\phi \qquad (5)$$

From Eq. (5), we can figure out that the PHE contribution has slope of $\sin^2\theta_M$ at fixed ϕ. Due to the $\sin 2\phi$ dependent term in Eq. (5) they have different slopes with different ϕ as seen in Fig. S3e. Figure S3f show the slope of Fig. S3e at each ϕ as function of $\sin 2\phi$ and the slope means PHE resistance. Using this method, we can not only extract AHE and PHE resistance with much smaller external magnetic field than anisotropy field, but also determine θ_M by Eq. (4). These extracted AHE resistance and PHE resistance have important role in analysis of harmonic Hall signals.

Supplementary Note 5: Contribution of Harmonic Hall result according to existence of $H_{K,2}$ consideration in spin-orbit torque analysis

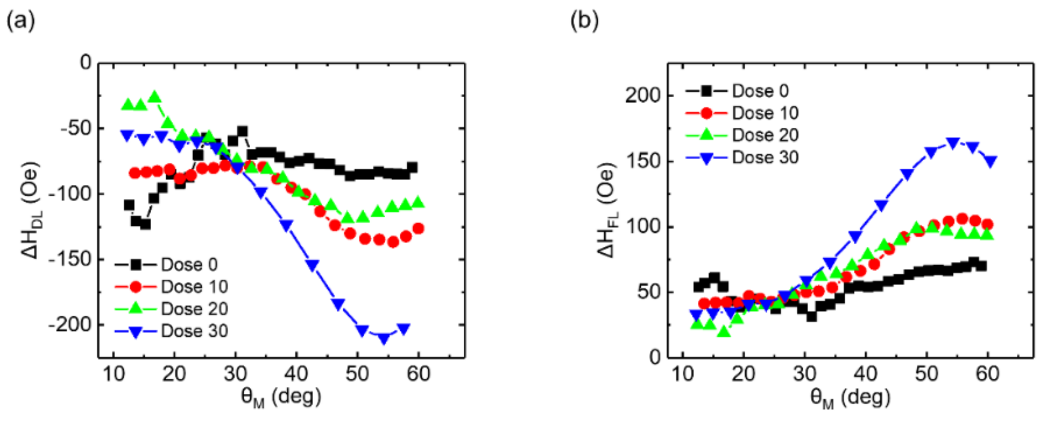

FIG. S5. Calculated effective field at various dose amounts without 2nd order PMA consideration. (a) DLT and (b) FLT effective field as function of θ_M.

The SOT analysis method using the 1st and 2nd order harmonic Hall signals with AC current is commonly utilized because of its ability to classify two different contributions of SOT and easy measurement process. At first many researches is conducted on much lower external magnetic field comparing anisotropy field, so only 1st order PMA effective field is under consideration [5,6,7]. However, as the studies on angular dependence of DLT and FLT effective field progress, it is also known that the 2nd order PMA field plays an important role in accurate analysis on harmonic Hall signals [4,8,9]. So, we consider only the 1st PMA on our data and show

the importance of contribution of 2nd PMA in the harmonic Hall signal analysis for accurate SOT induced effective field calculation. Because the Hall voltage follows,

$$V_H = V^{1\omega} \sin \omega t - V^{2\omega} \cos 2\omega t \qquad (6)$$

And here, $V^{1\omega}$ and $V^{2\omega}$ is the first order and the 2nd order harmonic Hall voltages, respectively, and expressed as,

$$V_x^{1\omega} = V_y^{1\omega} = V_{AHE}\sqrt{1-\left(\frac{H_{ext}}{H_K^{eff}}\right)^2} \qquad (7)$$

$$V_x^{2\omega} = \frac{V_{AHE}H_{ext}}{2\left(H_K^{eff}\right)^2}\left[\frac{\Delta H_{DL}}{1-\left(H_{ext}/H_K^{eff}\right)^2} - \Delta H_{FL}\right] \qquad (8)$$

$$V_y^{2\omega} = \frac{V_{AHE}H_{ext}}{2\left(H_K^{eff}\right)^2}\sqrt{1-\left(\frac{H_{ext}}{H_K^{eff}}\right)^2}\left[\frac{\Delta H_{FL}}{1-\left(H_{ext}/H_K^{eff}\right)^2} - \Delta H_{DL}\right] \qquad (9)$$

So, ΔH_{DL} and ΔH_{FL} can be calculated by measured $V^{1\omega}$ and $V^{2\omega}$ at each direction. The calculated DLT and FLT effective field can be rewritten as form like

$$\begin{pmatrix}\Delta H_{DL}\\ \Delta H_{FL}\end{pmatrix} = \frac{1}{B_0^2-A_0^2}\begin{pmatrix}-A_0 & B_0\\ -B_0 & A_0\end{pmatrix}\begin{pmatrix}T_x\\ T_y\end{pmatrix} \qquad (10)$$

and T_x, T_y, A_0 and B_0 is defined as

$$T_x \equiv -\frac{2V_x^{2\omega}}{\left(\partial V_x^{1\omega}/\partial H_{ext}\right)}\left(\frac{V_x^{1\omega}}{V_{AHE}}\right) \qquad (11)$$

$$T_y \equiv \frac{2V_y^{2\omega}}{\left(\partial V_y^{1\omega}/\partial H_{ext}\right)} \qquad (12)$$

$$A_0 \equiv 1 \qquad (13)$$

$$B_0 \equiv R\left(1-\left(\frac{H_{ext}}{H_K^{eff}}\right)^2\right) \qquad (14)$$

With above equations, we can replot the harmonic Hall voltage to DLT and FLT effective fields as function of θ_M, as shown in Fig. S4a and Fig. S4b. These results show quite different angular dependence at high tilting angle and the dose amount dependent tendency does not appear well at smaller angle case comparing with

case of considering 2nd order PMA. This suggests that the 2nd order PMA has a large enough influence on analysis of harmonic Hall signal at not only high angle magnetization tilting case, but also on calculation on effective fields in small tilting angle.

Supplementary Note 6: ρ_{xx} and ρ_{yx} of Platinum single layer with He^{+} ion irradiations

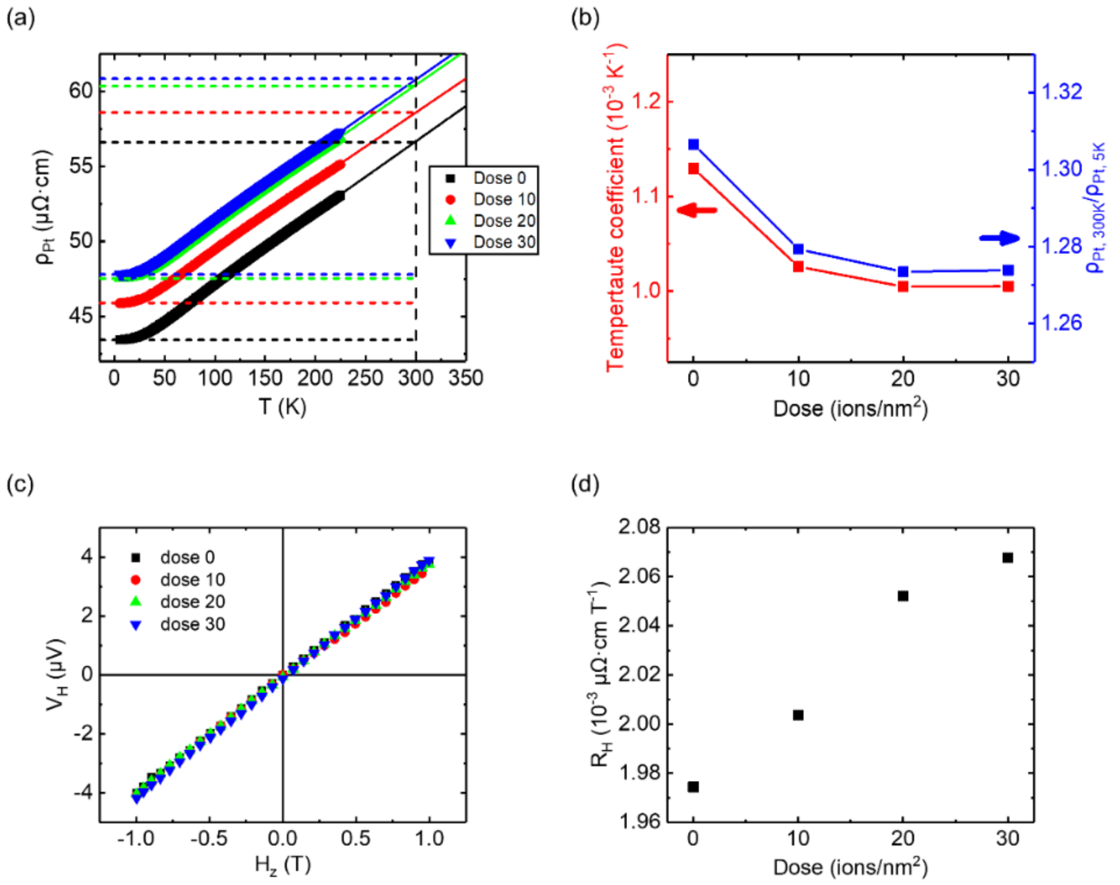

FIG. S6. Longitudinal and transverse resistivity changes by ion irradiation on single Pt layer. (a) Temperature dependent resistivity at various doses, here the solid line means linear fitted result excluding low temperature data, and (b) calculated value of temperature coefficient and RRR. (c) Field dependence of ordinary Hall effect using DC current with amplitude of 1 mA and (d) extracted Hall coefficient.

For understanding influence caused by ion irradiation induced Pt layer characteristic change, we measure the longitudinal and transverse resistivity by low-temperature resistance and ordinary Hall effect measurement, respectively. The Fig. S5a show the temperature dependent resistivity on Pt single layer from 5 K to 225 K and extracted temperature coefficient and residual-resistivity ratio (RRR) is seen in Fig. S5b. The temperature coefficient follows equation of $\rho = \rho_0(1 + \alpha_{Temp} \cdot (T - T_0))$ in linear resistivity region, here the α_{Temp} is

temperature coefficient, one can easily extract α_{Temp} using linear fitting. In RRR case, defined as ρ_{300K}/ρ_{5K} in here, we pick the resistivity at 5 K instead of 0 K for calculation of RRR because of measurement limitation and resistivity of 300 K is calculated extending with linear fitting as shown in FIG. S5a. Not only longitudinal resistivity, transverse resistivity also can be inferred with ordinary Hall effect (OHE) measurement. The measured Hall voltage and its Hall coefficient is shown in Fig. S5c-d, respectively. From this result, we can suggest that the ion irradiation process increases longitudinal and transverse resistivity both.

References

[1] Brown, W. F. Jr., Micromagnetics (Wiley, New York, 1963), Sect. 5.2.

[2] Okamoto, S. *et al*. Enhancement of magnetic surface anisotropy of Pd/Co/Pd trilayers by the addition of Sm. *J. Appl. Phys.* **90**, 4085 (2001).

[3] Okamoto, S. *et al*. Chemical-order-dependent magnetic anisotropy and exchange stiffness constant of FePt (001) epitaxial films. *Phys. Rev. B* **66**, 024413 (2002).

[4] Garello, K. *et al*. Symmetry and magnitude of spin-orbit torques in ferromagnetic heterostructures. *Nat. Nanotechnol.* **8**, 587-593 (2013).

[5] Kim, J. *et al*. Layer thickness dependence of the current-induced effective vector in Ta/CoFeB/MgO. *Nat. Mater.* **12**, 240-245 (2013).

[6] Hayashi, M. *et al*. Quantitative characterization of the spin-orbit torque using harmonic Hall voltage measurements. *Phys. Rev. B* **89**, 144425 (2014).

[7] Lee, H. -R. *et al*. Spin-orbit torque in a bulk perpendicular magnetic anisotropy Pd/FePd/MgO system. *Sci. Rep.* **4**, 6548 (2014).

[8] Qiu, X. *et al*. Angular and temperature dependence of current induced spin-orbit effective fields in Ta/CoFeB/MgO nanowires. *Sci. Rep.* **4**, 4491 (2014).

[9] Yun, S. J. *et al*. Accurate analysis of harmonic Hall voltage measurement for spin–orbit torques. *NPG Asia Materials* **9**, e449 (2017).
